# The intersection of depression and disability: A personal and professional perspective

**DOI:** 10.4102/sajpsychiatry.v30i0.2429

**Published:** 2024-12-11

**Authors:** Jean E. Augustyn

**Affiliations:** 1 Private, South Africa

Living with treatment-resistant depression (TRD) and occupational disability is tough.

Up until 8 years ago, I was a passionate nurse educator, specialised in emergency nursing. I had previously practised as a Professional Nurse, living on adrenaline in emergency situations (in-hospital, on ambulances both on the road and in the air). Emergency nursing and later training and development of other nurses were my passion.

I have lived with depression since high school days and was formally diagnosed in my twenties. Years later, it became TRD. I was using this antidepressant and then another, increasing dosages, changing and augmenting medications. I even tried transcranial magnetic stimulation (TMS). I was admitted to psychiatric clinics and ended up in Stikland Hospital when all my medical aid benefits were depleted.

Because nothing else worked, electroconvulsive therapy (ECT) was started. It was commenced while I was severely unwell. My employer started considering occupational disability. After a lousy 6 months, I was declared too unwell to continue my employment (despite my love for my nursing). My treating psychiatrist at the time suggested a gradual return to work, but this was not considered feasible by my former employer.

I wish I had known more about my rights and the consequences of prolonged sick leave and occupational disability during those last 6 months of employment. It is possible that I did know about my rights, or I was not well enough to research them or get advice, and by now, I cannot remember the details.

When the depression becomes too severe, I go back to the psychiatric clinic. This, of course, depends on what remaining benefits are available on my medical aid. When the medical aid benefits are depleted, I stay at home and pretend that all is fine. I try to ‘survive’. Sometimes, it ends up ugly: another crash or another suicide attempt.

The treatments continue (medications, ECTs, even ketamine infusions). The hospitalisations continue. After 21 days in-patient care, the prescribed medical benefits are depleted. That is it: the medical aid had spoken to say I should no longer receive care.

Last year, I went back to Stikland Hospital (again) and was able to stay for longer. The hospitalisation and group therapy certainly helped.

I struggle to understand how the prescribed minimum benefits (PMB) for depression stipulate only 21 days of in-patient care. There have been times when being admitted for 3 weeks was just not enough. How can medical aids pay for ‘unlimited’ hospital days in private hospitals for medical admissions but not for extended psychiatric admissions?

My memory has been affected. I will never again be able to function in my former professional role. There is so much that is just missing from my past. I struggle to remember passwords, decisions made, family events.

In my experience, the TRD and occupational disability have been feeding off one another, creating a vicious cycle. The continued disability worsens the depression. The depression just does not lift significantly, which then affects my functionality and deepens my disability.

My treating psychiatrist and psychologist are so graceful and have tried to do as much as possible with available resources. They have never given up on me and I truly appreciate it. It is probably very disconcerting for them to have a patient like me.

It has been 8 years since I wore my epaulettes. I still sit crying at my psychologist’s office when I talk about how I miss my profession and how useless I feel. He continues to listen and supports me and remains non-judgemental, patient, allowing me to express my feelings and frustrations.

I loved nursing and later, training people so that they could render proper emergency care. My occupational disability due to the TRD took it away. Or is it that the depression is made worse by the occupational disability? In the meantime, I am forced to ‘live’ with TRD and occupational disability.

I hope that by sharing my experiences, medical aids can see how they are letting down people with conditions like mine. I have also come to the conclusion that awareness around mental health at work is something that really needs to be recognised and spoken about.

The views and experiences in this article are reflective of my personal journey and should not be read as a piece of academic work.

